# Anti-Tumor Effects of Atractylenolide I Isolated from *Atractylodes macrocephala* in Human Lung Carcinoma Cell Lines

**DOI:** 10.3390/molecules181113357

**Published:** 2013-10-29

**Authors:** Huanyi Liu, Yajie Zhu, Tao Zhang, Zhenguo Zhao, Yu Zhao, Peng Cheng, Hua Li, Hui Gao, Xiaomei Su

**Affiliations:** Department of Oncology, Chengdu Military General Hospital, Chengdu 610083, Si Chuan Province, China; E-Mails: huanyiliucd@126.com (H.L.); yjzhucdh@yeah.net (Y.Z.); taozhangcdh@126.com (T.Z.); zgzhaocdh@163.com (Z.Z.); yuzhaocdh@yeah.net (Y.Z.); pengchengcdh@126.com (P.C.); hlicdh2013@126.com (H.L.); huigaocdh@126.com (H.G.)

**Keywords:** atractylenolide I, *Atractylodes macrocephala*, antitumor activity, apoptosis, lung cancer

## Abstract

Atractylenolide I (ATL-1) is the major sesquiterpenoid of *Atractylodes macrocephala*. This study was designed to investigate whether ATL-1 induced apoptosis in A549 and HCC827 cells *in vitro* and *in vivo*. In our results, ATL-1 significantly decreased the percentage of *in vitro* viability, in a dose-dependent manner. In addition, DAPI staining and flow cytometry tests demonstrated the induction of apoptosis by ATL-I. Western blot analysis indicated that the protein levels of caspase-3, caspase-9 and Bax were increased in A549 and HCC827 cells after ATL-I exposure; to the contrary, the expressions of Bcl-2, Bcl-XL were decreased after treatment with ATL-1. In the *in vivo* study, ATL-I effectively suppressed tumor growth (A549) in transplanted tumor nude mice with up-regulation of caspase-3, caspase-9, and Bax and down-regulation of Bcl-2 and Bcl-XL. In conclusion, our results demonstrated that ATL-I has significant antitumor activity in lung carcinoma cells, and the possible mechanism of action may be related to apoptosis induced by ATL-I via a mitochondria-mediated apoptosis pathway.

## 1. Introduction

Lung carcinoma is one of the leading malignant cancers with the greatest incidence and highest mortality rate [1]. Lung carcinoma is commonly classified as small cell lung cancer (SCLC) and non-small cell lung cancer (NSCLC), and NSCLC is the overriding type, accounting for approximately 80% of the total cases [2]. Currently, great improvements have been made in the diagnosis and treatment of lung cancers, but the 5-year survival rate is still less than 70% [3]. Apoptosis, otherwise known as programmed cell death, has been extensively investigated in the past decades, and apoptosis has been recognized as an ideal method of cancer therapy [4,5]. What’s more, effective and promising anticancer drugs with apoptotic effects are currently limited [5], therefore, it is important to search for new reliable therapeutic agents that can effectively induce cancer cell apoptosis to treat lung cancers.

*A. macrocephala* (also called *Baizhu* in Chinese), which belong to the Compositae family, has been traditionally used as an important crude drug to treat stomach diseases, digestive disorders, and anorexia [6]. Recently, *A. macrocephala* has been also reported to exhibit a variety of other activities, such as antitumor, anti-inflammatory, and antioxidant properties [4,6]. *A. macrocephala* is known to contain a large number of polyacetylenes, sesquiterpenoids, and flavones [4,6,7]. Atractylenolide I (ATL-I) is the major sesquiterpenoid of the rhizome of *A. macrocephala*, and shows a wide spectrum of pharmacological activities such as antiinflammatory, digestion promoting, and antioxidant effects [6,8]. Moreover, ATL-I has been reported to have significant cytotoxic activities on human promyeloleukemic HL-60 cells [4]. Currently, there are plenty of investigations reporting that natural sesquiterpenoids are interesting resources for the discovery of new anticancer agents [9,10], but no systematic investigations on the anti-tumor effects of ATL-I isolated from *A. macrocephala* have been published thus far. Our present study is designed to investigate the anti-tumor effects of ATL-I and its possible mechanism of action, which should provide significant date for the application of ATL-I to treat lung carcinoma in the clinic.

## 2. Results and Discussion

### 2.1. Cytotoxic Effect of ATL-I on A549 Cells

**Figure 1:**
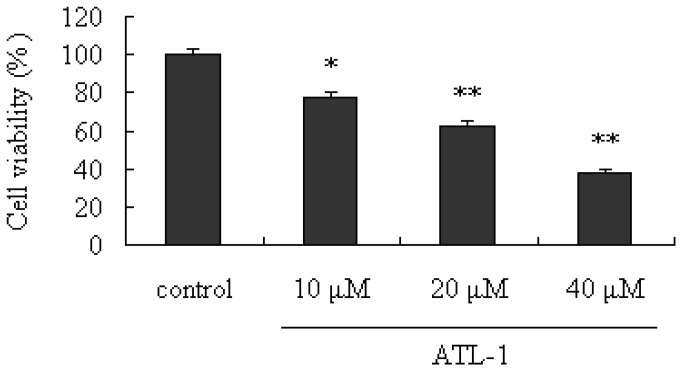
Cytotoxic effects of ATL-I on A549 cells. Cells were treated with ATL-I at the concentrations of 10, 20, and 40 μM for 48 hours, the cells viabilities were determined by the MTT assay), * *p* < 0.05, ** *p* < 0.01, *vs.* control.

To investigate the cytotoxic effects of ATL-I, the viability of A549 cells treated with ATL-I was determined by using the MTT assay. As shown in Figure 1, ATL-I exerted significant inhibitory effects on human lung carcinoma A549 cells (*p* < 0.05), compared with the control, in a concentration-response manner.

### 2.2. Effects of ATL-1 on Apoptosis in A549 Cells

**Figure 2:**
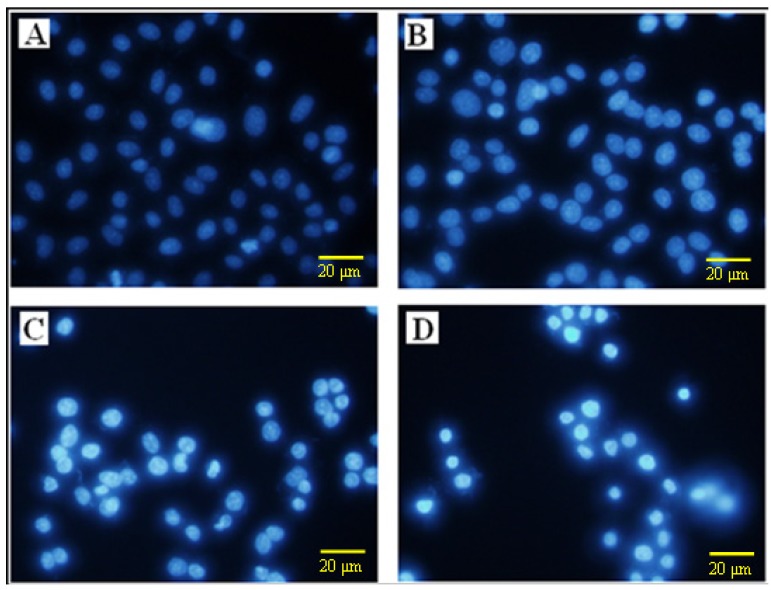
Determination of apoptosis in A549 cells by DAPI staining. Cells were treated with ATL-I at concentrations of 10, 20, and 40 μM for 48 hours, then cells were stained by DAPI and observed by fluorescence photomicrography (× 200). **A**–**D** represent the control, 10 μM, 20 μM, and 40 μM, respectively.

**Figure 3:**
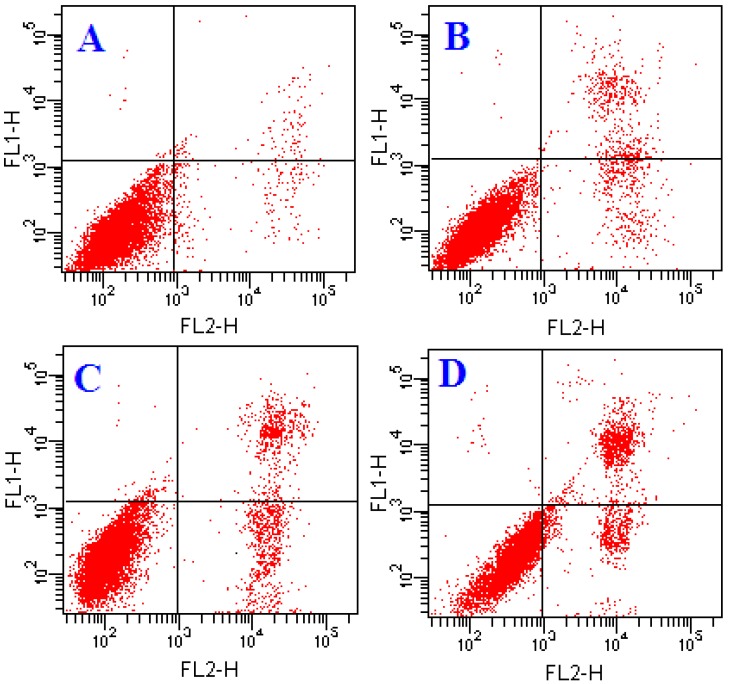
Determination of apoptosis in A549 cells by flow cytometry. Cells were treated with ATL-I at concentrations of 10, 20, and 40 μM for 48 hours, then cells were stained by Annexin V-FITC/PI and observed by flow cytometry. **A**–**D** represent the control, 10 μM, 20 μM, and 40 μM, respectively.

In order to further explore the induction of apoptosis by ATL-I in A549 cells, flow cytometry was performed and fluorescence photomicrographs were recorded after staining with Annexin-V/PI and DAPI, respectively. As can be seen from Figure 2, after treatment with ATL-I, a significant condensation of the nucleus was induced, which is characteristic of apoptosis. In addition, early apoptosis is characterized by translocation of phosphatidylserine (PS) from the inner layer of the plasma membrane to the outer surface, and Annexin-V can specially bind to PS and has commonly been used for determination of apoptotic cells [5,9]. In our present study, Annexin-V/PI staining was performed to evaluate apoptotic cells followed by treatment with 10, 20 and 40 μM concentrations of ATL-I in A549 cells, and the results demonstrated that ATL-I can induce significant apoptosis in A549 in a concentration-dependent manner (Figure 3).

### 2.3. ATL-I Induces Apoptosis in A549 and HCC827 Cells via a Mitochondria-Mediated Apoptosis Pathway

**Figure 4:**
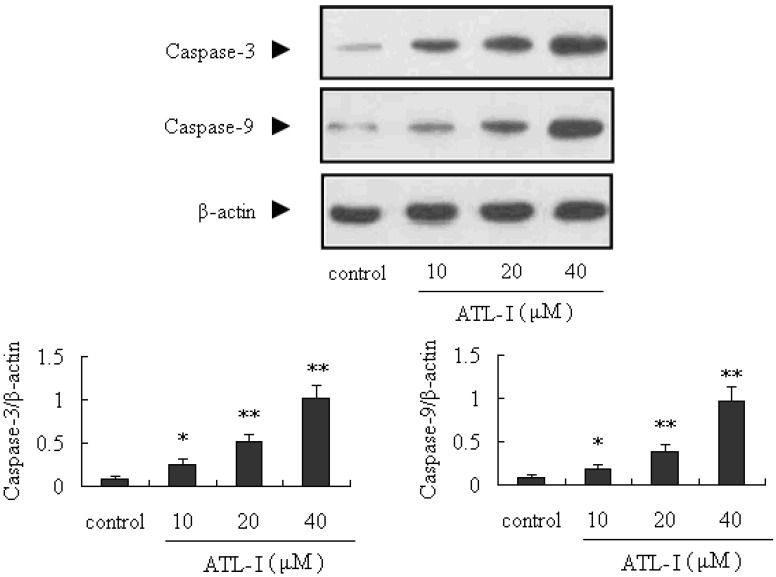
Effect of ATL-I on caspase-3 and caspase-9 expressions in A 549 cells. Cells were treated with ATL-I at the concentrations of 10, 20, and 40 μM for 48 hours, and the whole cell lysates were determined by western blot analysis using antibodies for caspase-3 and caspase-9 as indicated. All data were expressed as mean ± SEM (n = 3), * *p* < 0.05, ** *p* < 0.01, *vs.* control.

Apoptosis is very important for the development and health of multicellular organisms, and is a cell-intrinsic programmed suicide mechanism for damaged cells [4,5,11,12]. Mitochondria-mediated intrinsic apoptosis is one of the major apoptotic pathways. What’s more, caspase proteins (cysteine-aspartic acid proteases) are crucial mediators of apoptosis, and active caspase-3 is a marker for cells undergoing apoptosis. It’s well known that caspase-3, which is one of the key activated death proteases, can be commonly activated by caspase-9 [13,14]. In our present study, the caspase-3 and caspase-9 proteins were significantly up-regulated by treatment with ATL-I (*p* < 0.05), in a concentration-dependent manner (Figure 4). 

**Figure 5:**
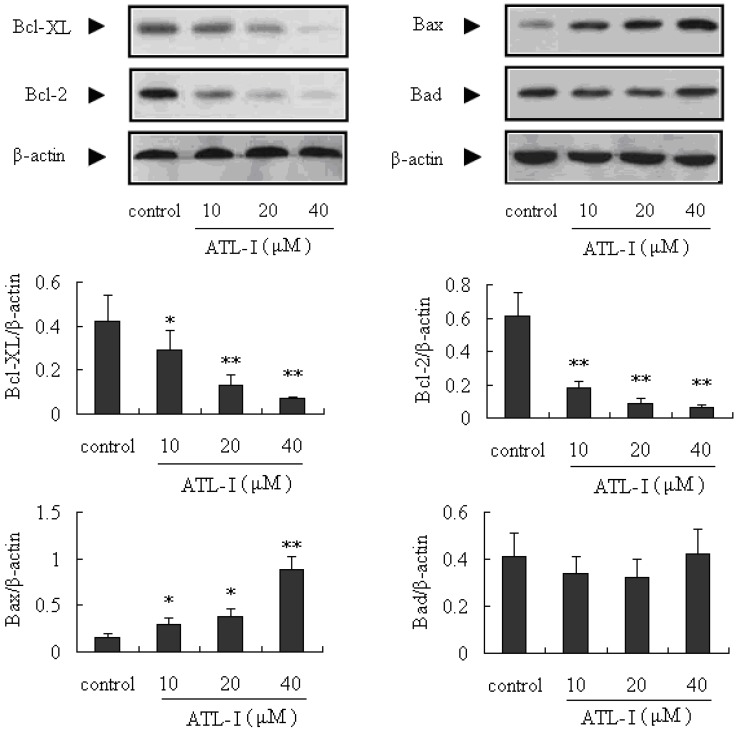
Effect of ATL-I on Bcl-2 family proteins (Bcl-XL, Bcl-2, Bax, and Bad) expressions in A 549 cells. Cells were treated with ATL-1 at the concentrations of 10, 20, and 40 μM for 48 hours, and the whole cell lysates were determined by western blot analysis using antibodies for Bcl-XL, Bcl-2, Bad, and Bad as indicated. All data were expressed as mean ± SEM (n = 3), * *p* < 0.05, ** *p* < 0.01, *vs.* control.

Bcl-2 family is another kind of important protein in the mitochondria-mediated intrinsic apoptosis. Currently, the Bcl-2 family proteins have generated significant attention as potential apoptosis regulators in cancer treatment [15,16]. Bax and Bad are the well-known pro-apoptotic proteins, and Bcl-2 and Bcl-XL are anti-apoptotic proteins, so the ratio of Bax and Bad/Bcl-2 and Bcl-XL has a key role in the development of apoptosis. An increased ratio can activate the mitochondria-mediated apoptotic cell death pathway [17,18]. In our present study, we determined the expressions of Bcl-XL, Bcl-2, Bax, and Bad proteins in A549 cells after treatment with ATL-I by the western blot method. Our results indicated that ATL-I (at concentrations of 10, 20, and 40 μM) can significantly down-regulate the expressions of Bcl-2 and Bcl-XL (*p* < 0.05), and significantly up-regulate (*p* < 0.05) the expression of Bax, in a concentration-dependent manner (Figure 5). In addition, the ratio of Bax and Bad/Bcl-2 and Bcl-XL also increased after treatment with ATL-I.

**Figure 6:**
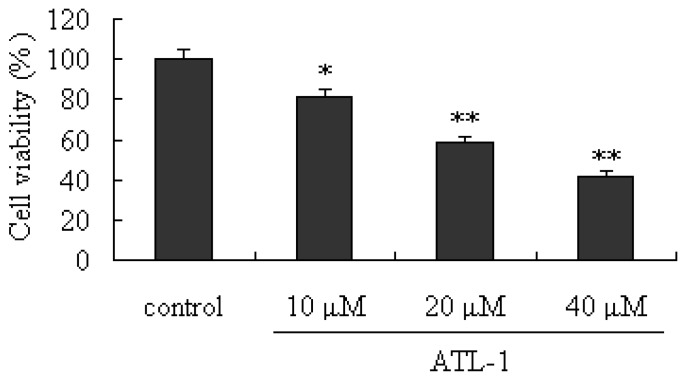
Cytotoxic effects of ATL-I on HCC827 cells. Cells were treated with ATL-1 at the concentrations of 10, 20, and 40 μM for 48 hours, the cells viabilities were determined by the MTT assay, * *p* < 0.05, ** *p* < 0.01, *vs.* control.

**Figure 7:**
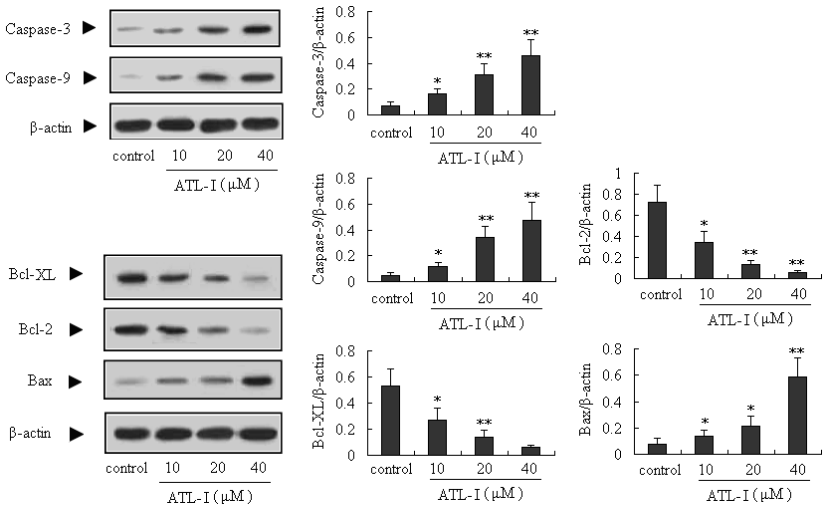
Effect of ATL-I on caspase-3, caspase-9, Bcl-XL, Bcl-2, and Bax expressions in HCC827 cells. Cells were treated with ATL-1 at concentrations of 10, 20, and 40 μM for 48 hours, and the whole cell lysates were determined by western blot analysis using antibodies for caspase-3, caspase-9, Bcl-XL, Bcl-2, and Bax as indicated. All data were expressed as mean ± SEM (n = 3), * *p* < 0.05, ** *p* < 0.01, *vs.* control.

To determine whether ATL-I-induced deaths observed in A549 cells can also occur in other lung carcinoma cell lines, parallel studies were performed on HCC827 cells. Exposure of HCC827 cells to ALT-I for 48 hours resulted in an obvious inhibitory effect (Figure 6). In addition, the results of our present study also indicated that ALT-I at the tested concentrations (10, 20, and 40 μM) can up-regulate the expressions of caspase-3, caspase-9 and Bax (*p* < 0.05), and down-regulate the expressions of Bcl-2 and Bcl-XL (*p* < 0.05) (Figure 7). All the results above indicated that ATL-I can induce apoptosis in lung carcinoma cell lines, and the mitochondria-mediated apoptosis pathway may be one of the most important mechanisms involved in this activity.

### 2.4. Antitumor Activity of ATL-1 on Nude Mice

**Figure 8:**
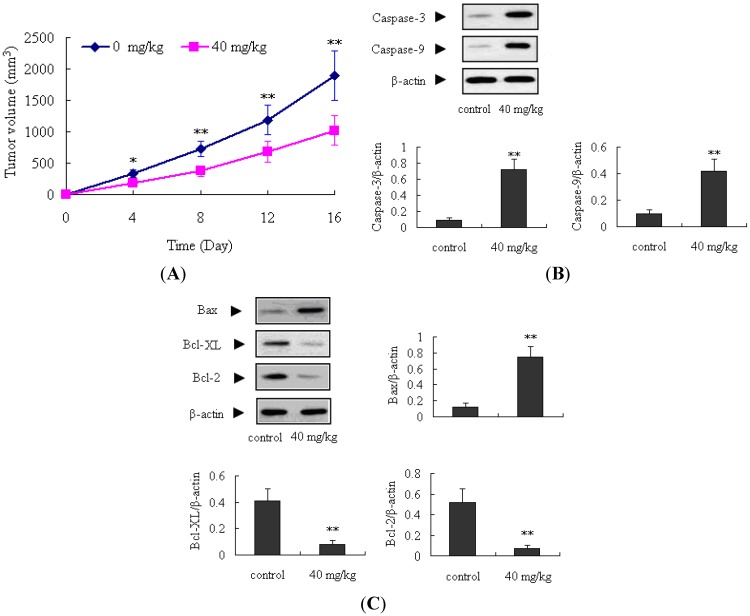
Antitumor activity of ATL-I in A549 cells *in vivo*. (**A**) The effect of ATL-1 on tumor volume in 16 days observation (n = 10). (**B**) Expressions of caspase-3 and caspase-9 in the tumor tissues (n = 3). (**C**) Expressions of Bax, Bcl-2 and Bcl-XL in the tumor tissues (n = 3). All data were expressed as mean ± SEM, * *p* < 0.05, ** *p* < 0.01, *vs.* control.

The *in vivo* antitumor effects of ATL-1 on A549 cells were further evaluated in a transplanted tumor nude mice model. Tumor growth was significantly inhibited after treatment with ATL-I at a dose of 40 mg/kg during the 16 days of observation, compared to the control mice (*p* < 0.05). Additionally, the expressions of caspase-3, caspase-9, and Bax proteins in the ATL-1 treated mice were obviously increased compared with the control mice (*p* < 0.01); on the contrary, the Bcl-2 and Bcl-XL proteins were decreased (*p* < 0.01) (Figure 8). These findings confirmed the antitumor activity of ATL-I in A549 cells, and also confirmed that the apoptosis induced by ATL-I *in vivo* is associated with the mitochondria-mediated apoptosis pathway.

## 3. Experimental

### 3.1. Chemicals

Silica-gel was purchased from Qingdao Haiyang Chemical Co., Ltd. (Qingdao, China). MeOH (AR), petroleum ether (AR), ethyl acetate (AR), *n*-butanol (AR) were purchased from Sinopharm Chemical Reagent Co., Ltd. (Shanghai, China); Sephadex LH-20 was purchased from H&E Co., Ltd. (Beijing, China). The RPMI 1640 media and fetal bovine serum (FBS) were purchased from Invitrogen (Carlsbad, California, CA, USA). 3-(4,5-Dimethylthiazol-2-yl)-2,5-diphenyltetrazolium bromide (MTT), DAPI and dimethyl sulfoxide were purchased from Sigma (St. Louis, Missouri, MO, USA). Human caspase-3, caspase-9, Bcl-XL, Bcl-2, Bax, Bad monoclonal antibody, and Annexin V/FITC kit was purchased from Beyotime (Jiangsu, China).

### 3.2. Animals

BALB/C nude mice (5~6 weeks old) were purchased from the Vital River Laboratories (Beijing, China). The experimental protocols were approved by the Animal Care and Use Committee of our hospital.

### 3.3. Isolation and Preparation of ATL-1

Dried rhizome of *A. macrocephala* was purchased from Tong-ren-tang Pharmaceutical Group, and a voucher specimen (S1206-12#) was deposited at our laboratory. The powdered *A. macrocephala* (50 kg) extracted three times with 75% aqueous ethanol (5 times of the materials) by reflux (each extraction period lasted 3 hours). The solvent was evaporated under vacuum to afford the crude total extract of *A. macrocephala*. The extract was then suspended in water and successively partitioned with petroleum ether, ethyl acetate, and water-saturated *n*-butanol. The ethyl acetate fraction was subjected to repeated column chromatography over silica gel (100–200 mesh) eluting with petroleum ether–acetone (15:1~1:1). The combination of similar fractions on the basis of TLC analysis afforded six fractions I-VI. By using a series of chromatographic techniques, such as silica gel column chromatography (200–300 mesh) and Sephadex LH-20 chromatography, a compound (500 mg) was isolated from fraction II and identified as atractylenolide I by comparing its NMR data with the literature [19].

### 3.4. Cell culture

Human lung cancer cell line A549 was purchased from the American Type Culture Collection (Manassas, Virginia, VA, USA). The cells were cultured in RPMI-1640 medium with 10% fetal bovine serum and antibiotics (100 U/mL penicillin and 100 μg/mL streptomycin). The cell line was kept at 37 °C in 5% CO_2_/95% air.

### 3.5. MTT Reduction Assay

Cells (1 × 10^4^/0.2 mL) were seeded in 96-well plates and treated on the following day with indicated concentrations of ATL-I for 48 hours. After that, MTT assay was carried out using the standard protocol and the optical density (OD) was read at 570 nm using a 96-well plate reader. Since reduction of MTT only occurred in metabolically active cells, the level of activity was a measure of the viability of the cells. The inhibition rate was calculated according to the following formula: 

(OD_control_ – OD_treatment_)/OD_control_ × 100%.

### 3.6. Apoptosis Assay

Two methods were used for apoptosis assays. Firstly, A549 Cells (5 × 10^5^/mL) were seeded in 6-well plates. A549 cells were treated with 10, 20, 40 μM of ATL-I for 48 hours, then cells were washed with PBS and stained using an Annexin V/FITC kit. The cell apoptosis was detected by flow cytometry (FCM) on a FACScalibur flow cytometer (BD Biosciences, San Jose, California, CA, USA). Secondly, the cells (1 × 10^4^/0.2 mL) were seeded in 96-well plates and then treated with 10, 20, 40 μM of ATL-I for 48 hours, then cells were stained by DAPI and examined and photographed by using a fluorescence microscope (BX41-32PO2-FLB3, Olympus, Tokyo, Japan).

### 3.7. Western Blot Analysis

Total proteins of cells or tumor tissues were extracted, and then equal amounts of protein (40 μg) were separated by sodium dodecyl sulfate/polyacrylamidegel electrophoresis (SDS/PAGE), blotted on polyvinylidene difluoride (PVDF), and probed with corresponding monoclonal antibody, and subsequently with goat anti-rabbit/HRP, and detected by chemiluminescence. To measure protein loading, antibodies directed against β-actin were used.

### 3.8. *In Vivo* Antitumor Efficacy Study

A549 cells (3 × 10^6^/0.2 mL per mouse) were suspended in sterile PBS and injected subcutaneously into the right flank of the mice. Mice were randomly divided into two groups (10 mice per group). When the tumors grew to approximate 2–3 mm in diameter, the treatment group mice received ATL-I (40 mg/kg, intraperitoneally injection for 16 days). The control group received an equal volume of solvent control (1% DMSO). Tumor sizes were measured every four days. Tumor volumes were determined by a caliper and calculated according to the previously reported formula [20]: 

Volume = (width^2^ × length)/2 (1)

All animals were killed immediately after 16 days of drug exposure, and the tumors tissues were collected for western blot determination.

## 4. Conclusions

In conclusion, our results demonstrated that ATL-I has significant antitumor activity on A549 and HCC827 cells, and the possible mechanism of action may be related to apoptosis induced by ATL-I via a mitochondria-mediated apoptosis pathway. Therefore, ATL-1 may be an interesting potential apoptosis inducer that could be a candidate for the treatment of lung cancer in the future. However, more laboratory investigations are necessary for its safety evaluation and elucidating the complete mechanism.
